# Acute Abdomen Requiring 14 Relooks and Five Months in ICU With a Favourable Outcome: A Report and Review of Literature

**DOI:** 10.7759/cureus.114748

**Published:** 2026-08-18

**Authors:** Umme Nashrah, Ahmad Mandoob Salameh Obeidat, Umm E Amara, Nissar Shaikh

**Affiliations:** 1 Medicine, Deccan College of Medical Sciences, Hyderabad, IND; 2 Anesthesiology and Critical Care, Hamad Medical Corporation, Doha, QAT; 3 Obstetrics and Gynaecology, Deccan College of Medical Sciences, Hyderabad, IND; 4 Surgical Intensive Care, Hamad Medical Corporation, Doha, QAT

**Keywords:** acute abdomen, advance hemodynamic monitoring, aki, antimicrobials, bogota bag, bowel oedema, cinma, enteral and parenteral nutrition, fluid resuscitation, invasive ventilation

## Abstract

An open abdomen or laparostomy is a lifesaving strategy used in critical situations to protect vital organs and stabilize patients before definitive closure of the abdominal incision. The indications for maintaining an open abdomen are broadly classified into two categories: the first is where it is impossible to close the abdomen due to bowel oedema and abdominal compartment syndrome (ACS), and the second is where there is a need for damage-controlled laparotomy or repeated intra-abdominal interventions. These open abdomens are frequently reported to require up to four relook laparotomies, and the open abdomen is commonly closed within 10 days. There are no reports in the literature regarding upper limits for relook relaparotomies or maximum duration of an open abdomen with a favourable outcome. We report a case of acute abdomen and severe bowel oedema, with the abdomen unable to be closed and kept open with a Bogota bag, requiring 14 laparotomies and multiple bouts of sepsis, requiring five months of intensive care therapy with a favourable outcome.

## Introduction

Damage control open abdomen (OA) therapy has become increasingly common in the management of critically ill surgical patients. However, this technique is associated with significant complications, including enterocutaneous and entero-atmospheric fistulas, sepsis, and ventral hernias [1]. Patients undergoing OA therapy often require prolonged intensive care, placing them at increased risk of sepsis, morbidity, and mortality [2]. Risk factors for keeping the abdomen open after exploration are systemic factors such as higher severity of disease or trauma, profound shock, and elderly patients, whereas the local factors include severe bowel edema, persistent and recurrent abdominal infections, sepsis, entero-atmospheric fistulas, and a higher number of explorations [3]. Early fascial closer after laparotomies are very important, as it preserves the abdominal anatomy, lowers complications and thus causing decrease in morbidity and mortality. The survival rate after multiple laparotomies and a prolonged period with an open abdomen is rarely reported in the literature [4]. We report a case a young patient with an acute abdomen, where it was not possible to close the abdomen after first exploration; the patient subsequently underwent 14 laparotomies for relooks requiring 5 months of acute and chronic intensive care therapy.

## Case presentation

A 28-year-old obese male with a recently diagnosed type 2 diabetes mellitus on oral hypoglycaemics presented to the emergency department with severe epigastric pain, which had been increasing in intensity for the last two days without any aggravating or relieving factors, hematemesis or haematochezia. He had made three visits to the emergency clinic for milder abdominal pain and had been treated with antacids and analgesia.

On examination, he appeared critically ill with tachycardia (120-134 beats/min), tachypnoea (26-30 breaths/min), borderline low blood pressure (95/54 mmHg), abdominal distension, and generalized tenderness. Laboratory investigations showed leukocytosis (27.5 × 10³/µL), elevated renal parameters (blood urea nitrogen 35.4 mmol/L, serum creatinine 428 µmol/L), and elevated inflammatory markers (C-reactive protein 357 mg/L, procalcitonin 14.4 ng/mL) (Table 1). During the examination, his blood pressure dropped, and he became hemodynamically unstable. He was clinically diagnosed with an acute abdomen and septic shock, started on broad-spectrum antibiotics (Tazocin®) after taking cultures, and immediately taken for an exploratory laparotomy. The laparotomy revealed six liters of pus in the peritoneal cavity, a perforation with gangrenous patch in the jejunum and severe bowel oedema. The affected bowel was resected, thorough peritoneal lavage was performed, and temporary abdominal closure with a Bogota bag was used because primary closure was not possible. The Bogota bag used in this case was a sterilized 2-litre Ringer Lactate bag sewn to the skin of the anterior abdominal wall.

**Table 1:** Patient's laboratory values and their reference ranges

Parameter	Patient's values	Reference range
White Blood Cells	27.5 × 10³/µL	4 x to 11 x 10³cells/µL
Blood Urea Nitrogen	35.4 mmol/L	2.1 to 8.5 mmol/L
Serum Creatinine	428 µmol/L	62 to 115 µmol/L
C-Reactive Protein	357 mg/L	<10 mg/L
Procalcitonin	14.4 ng/mL	< 0.05 ng/mL to 0.1 ng/mL
Serum Lactate	8.1 mmol/L	0.5-1 mmol/L

**Figure 1:**
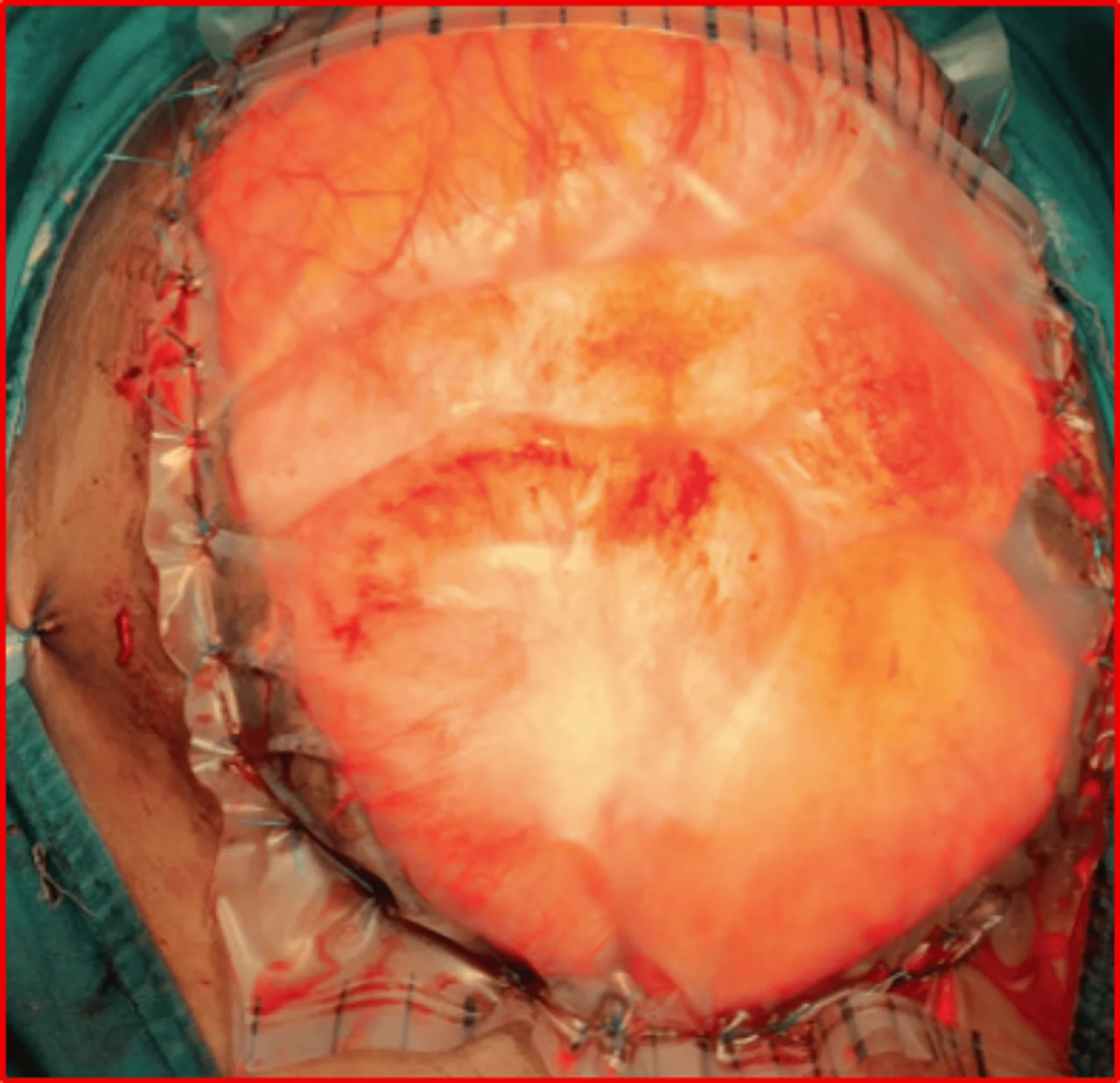
Bogota bag for temporary abdominal closure

Post-operatively, the patient was transferred to the Surgical Intensive Care Unit (SICU) where he was intubated, ventilated and required high doses of noradrenaline and vasopressin to maintain hemodynamics. His serum lactate was high (8.1 mmol/L). Resuscitation was continued with fluids guided by pulse-contoured continuous cardiac output (Picco®) and advanced hemodynamic monitoring. On day 2, he was taken for a relook laparotomy, and peritoneal lavage was performed, along with small bowel anastomosis, and the abdomen was kept open with a Bogota bag (Figure 1). Post-operative resuscitation was continued, his kidney function improved, and he was weaned off vasopressin and started on TPN. He remained sedated and on controlled mechanical ventilation.

He underwent a second relook laparotomy on day 4, due to pus being visualised through the Bogota bag, during which peritoneal lavage was performed, oedematous bowel was seen, and the abdomen was again closed with Bogota bag. He stabilized and was weaned off from noradrenaline. On day 8, he developed a fever and greenish fluid was seen through the Bogota bag. He underwent a third relook laparotomy and a bile leak from anastomosis was seen. The anastomosis was taken down, edges were refashioned, reanastomosis was done using a hand-sewn technique, and a feeding jejunostomy tube was inserted. Back in the SICU, he did not improve and required vasopressors. On day 10, greenish-yellow colored fluid was seen through the Bogota bag, and in the context of new sepsis and high sepsis markers, the patient was taken for a fourth relook laparotomy where leakage from the Jeju-jejunal anastomosis and slippage of the jejunal feeding tube were seen. Abdominal lavage was performed, the jejunal loop was fixed to the lateral abdominal wall, a Malecot catheter was inserted in the proximal jejunal loop, and a Bogota bag was reapplied. On day15, he became febrile (38.9°C), his vasopressor needs increased, and his antibiotics were escalated to meropenem, vancomycin, and anidulafungin on account of worsening sepsis markers (Table 2).

**Table 2:** Timeline of events including reasons of laparotomies, sepsis episodes, microbiology and antibiotics SVC: Superior vena cava, TPN: Total parenteral nutrition, MODS: Multiorgan dysfunction.

Timing	Interventions	Septic Shock, MODS & Vasopressors	Microbiology	Antimicrobial and Duration
Admission	Exploratory laparotomy, bowel resection, Peritoneal lavage and insertion of Bogota Bag	Yes, and on noradrenaline and vasopressin. Ventilated, Acute kidney injury (AKI)	Cultures taken	Tazocin®
Day2	Reexploration, small bowel anastomosis, peritoneal lavage and Bogota bag application	Yes, and on noradrenaline and vasopressin. Ventilated	E. coli	Tazocin®
Day4	Relook laparotomy, peritonitis, bowel edema, peritoneal lavage and Bogota bag application	Yes, and on noradrenaline. AKI resolved. Ventilated TPN started.	None	Tazocin®
Day8	Reexploration, biliary leak, Reanastomosis done, peritoneal lavage and Bogota bag application	Yes, and on noradrenaline. Ventilated	E.coli and anaerobes	Meropenem, vancomycin and anidulafungin
Day10	Relook laparotomy, peritoneal lavage, jejunal feeding tube, Malecot drain insertion, Bogota bag application	Yes, and on noradrenaline. Ventilated	None	Meropenem, vancomycin and anidulafungin
Day15	Relaparotomy, insertion of 2^nd^ Malecot drain, peritoneal lavage, Bogota bag application	Yes, and on noradrenaline. Ventilated	None	Meropenem, vancomycin and anidulafungin.
Day17	None	No, off vasopressor, ventilated, enteral feeds started, remained on TPN	None	Meropenem, vancomycin and anidulafungin
Day19	Relook and malecot drains adjustments. peritoneal lavage, Bogota bag application	No, off vasopressors, ventilated, enteral feeds and TPN off	None	Meropenem, metronidazole and anidulafungin
Day22	Tracheostomy	Enteral feeds and ventilated	None	Meropenem, metronidazole and anidulafungin
Day41	None	Enteral feeds, off ventilation, supportive care	None	None
Day61	Reexploration, peritoneal lavage, Bogota bag changed	Septic shock, noradrenaline ventilated	Pseudomonas aeruginosa	Tazocin® for 5 days
Day72	Trachea was decanulated	Enteral feeds, supportive care	None	None
Day80	Tunneled central venous catheter inserted	Enteral feeds, supportive care removed, all invasive lines removed	None	None
Day86	Reexploration, multiple collections, replacement of Malecot catheters, peritoneal lavage, Bogota bag	Septic shock, vasopressors and ventilated, AKI, septic cardiomyopathy.	Pseudomonas aeruginosa MDRO	Meropenem
Day90	None	Off vasopressor, remained ventilated	None	Meropenem
Day110	Reexploration, multiple collections, replacement of malecot catheters, peritoneal lavage, Bogota bag	Septic shock, vasopressors and ventilated	None	Tazocin®
Day112	Reexploration, multiple collections, peritoneal lavage, Bogota bag	Septic shock, vasopressors and ventilated	None	Tazocin®
Day115	Reexploration, multiple collections, replacement of Malecot catheters, peritoneal lavage, Bogota bag	Septic shock, vasopressors and ventilated	None	Tazocin®
Day115		Critical illness neuromuscular abnormalities, blood sugar control, enteral feeding and supplementary.	None	Tazocin®
Day127		Off ventilation, recovered organ functions. Continued supportive care	None	None
Day144	Reexploration, removed slipped Malecot catheters, inserted new Malecort drains, excision enteric fistula, peritoneal lavage, Bogota bag	Septic shock, ventilated	E Coli(ESBL)	Meropenem (7 days)
Day173	SVC obstruction syndrome	Therapeutic anticoagulation	None	None
Day280	Laparotomy, Bogota bag removed, excision of enteric fistula, mesh repair, abdominal closure	Supportive care	None	Cefazoline single dose
Day90	Transferred to surgical ward			
Day308	Discharged home			

**Figure 2:**
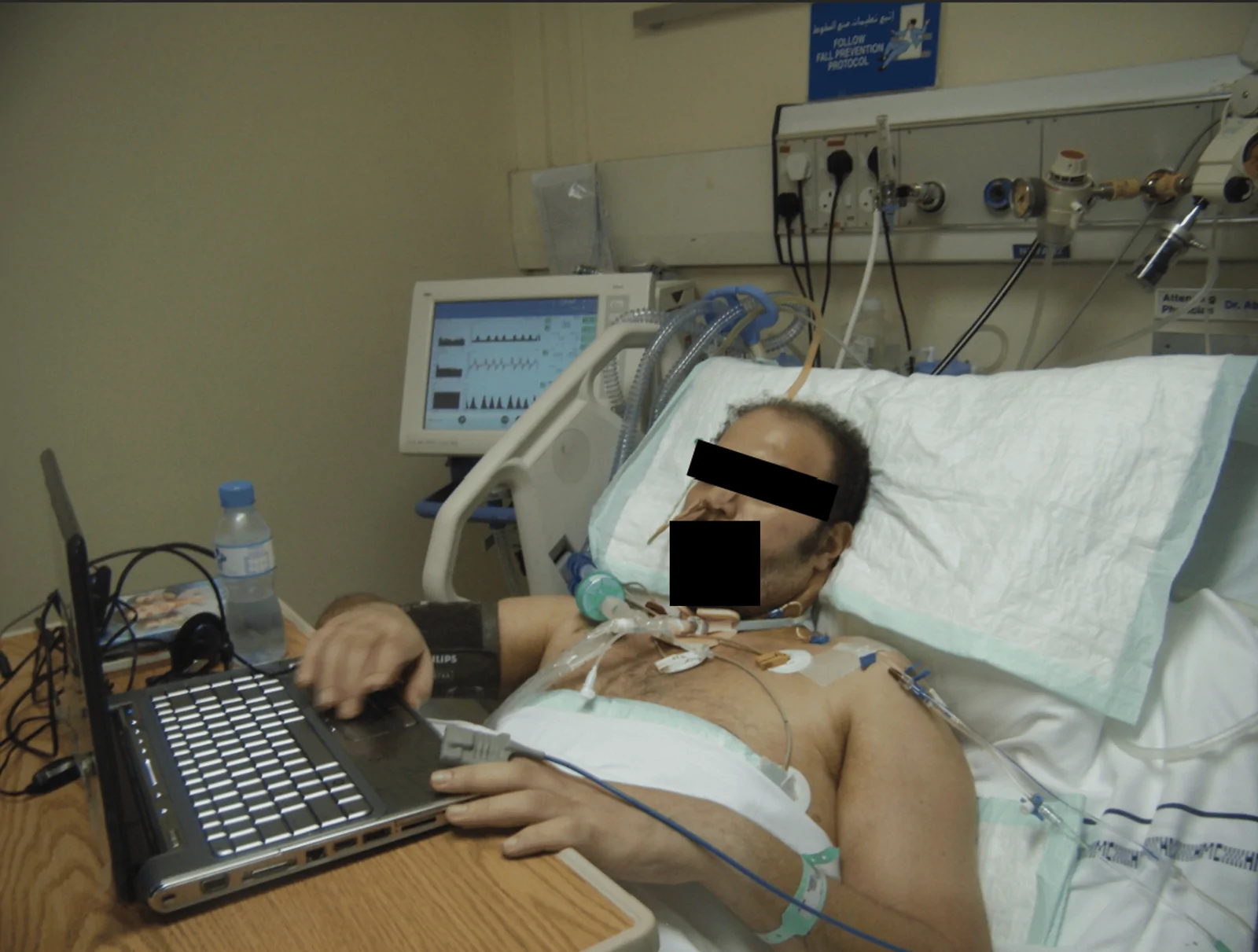
Patient playing on his laptop

He had a fifth laparotomy due to purulent fluid in the Bogota bag, during which a thorough peritoneal lavage was done, a second Malecot tube inserted in the proximal jejunal tube, the first Malecot catheter fixed, a feeding jejunostomy tube inserted, and the Bogota bag reapplied. He stabilised and was started on enteral feeds through the jejunostomy tube and weaned off TPN. He had a sixth exploration on day 19 for adjustment of both Malecot drains and underwent tracheostomy on day 22. He was weaned off sedation and maintained on low-dose remifentanil. He was awake, alert, and following commands, and was even able to use a laptop (Figure 2). His clinical condition continued to improve with enteral feeds and comprehensive intensive care support. By day 41, he had been successfully weaned to a tracheostomy cannula, and antimicrobial therapy was discontinued. His abdomen remained open with a Bogota bag in place, and the bowel oedema was gradually resolving.

On day 61, he developed a fever, worsening sepsis markers, and accumulation of pus-coloured fluid beneath the Bogota bag. He underwent a seventh relook laparotomy with peritoneal lavage. Postoperatively, his condition improved, allowing weaning from vasopressors and mechanical ventilation while continuing to tolerate enteral nutrition. He remained awake and stable, and his tracheostomy cannula was removed on day 72. On day 80, a tunneled central venous catheter was inserted, and all other invasive vascular access lines were removed.

However, on postoperative day 86, he developed sepsis, necessitating an eighth exploratory laparotomy with abdominal lavage. Following surgery, he required reinstitution of mechanical ventilation and vasopressors. He subsequently underwent a ninth relook laparotomy with abdominal lavage, during which the existing Malecot catheters were removed and replaced. Following these interventions, the patient's condition improved, enabling successful weaning from vasopressors while he remained on mechanical ventilation and enteral feeds. On day 110, he developed recurrent sepsis with septic shock, requiring a tenth exploratory laparotomy with abdominal lavage and adjustment of the Malecot drains. He subsequently underwent eleventh and twelfth exploratory laparotomies on postoperative days 112 and 115, during which the feeding jejunostomy tube was reinserted. Following these interventions, the patient was weaned off vasopressors and was found to have critical care-induced neuromuscular abnormalities (CINMA) resulting in the need for pressure support ventilation (PSV). Glycaemic control and nutritional status were optimised with enteral nutrition supplemented by total parenteral nutrition (TPN), with close biochemical and clinical monitoring. He was weaned off the ventilator and back on a tracheal cannula (Table 2). He was awake and stable, tolerated enteral feeds and demonstrated improving muscular power and movement. He recovered completely from acute kidney injury (AKI) and septic cardiomyopathy. He was transferred to the surgical ward and continued to improve (Table 2).

On day 144, one of the Malecot catheters slipped, resulting in leakage into the Bogota bag requiring a 13th exploratory laparotomy with reinsertion of the Malecot drains. Postoperatively, he was transferred to the SICU. On day 173, he developed superior vena cava (SVC) obstruction syndrome due to SVC thrombus related to the tunneled central venous catheter. He was treated with therapeutic low-molecular-weight heparin (LMWH), with subsequent improvement. By day 236, the abdominal wound had developed healthy granulation tissue beneath the Bogota bag, and he was tolerating an oral diet. On day 280, he underwent a 14th laparotomy involving removal of the Bogotá bag, excision of multiple small bowel fistulas, bowel resection and anastomosis, and definitive abdominal wall closure using mesh reinforcement.

Following the procedure, he continued to improve. He remained awake and stable and was successfully weaned off the ventilator. He was transferred to the surgical ward on day 290 while on an oral diet. He was decannulated on day 301, and he was discharged home on day 308. At follow-up visits in the outpatient surgical clinic at one, three, and six months after discharge, he remained well, with complete recovery of both physical and cognitive functions. He had returned to work as a computer engineer and had regained full functional independence.

## Discussion

The open abdomen technique (OAT), used to treat septic peritonitis and first described by McCosh in 1897, is still successfully used [5]. OAT has more recently been popularized by damage control surgery and laparotomies, mainly in trauma patients. OAT has two indications: the first is where, due to intra-abdominal pathologies, once a laparotomy is done, the abdomen cannot be closed, more frequently, as in our patient due to severe bowel oedema due to septic peritonitis; the second indication for OAT is damage controlled laparotomies, abdominal compartment syndrome, acute severe pancreatitis where it may be necessary to enter and exit the abdomen multiple times to achieve the interventional therapeutic goals [6]. There are multiple techniques to keep the abdomen open; our patient was managed with the Bogota bag, which has the advantage of allowing direct monitoring of the abdominal contents and facilitating early interventions while decreasing the need for repeated imaging studies [7]. Nowadays, the Bogota bag is used initially, and once the patient is stabilized, a vacuum-assisted (VAC) closed dressing is applied [8].

An open abdomen (laparostomy) is a temporary life-saving measure used in critically ill patients. Ideally, the fascia is closed within 48 to 72 hours. If early closure isn't possible, fascial closure is best achieved within 5 to 8 days. Delays beyond this window make primary closure significantly more difficult. The longer the abdomen remains open, the more the abdominal wall muscles and fascia retract and stiffen. This makes closing the muscles back together physically impossible, prompting the need for gradual tension devices or subsequent hernia repair [9]. In 2005, a landmark publication by Miller et al. reviewed 344 trauma patients who required an open abdomen and temporary abdominal coverage [9]. The study found a 25% wound complication rate (infections, abscesses, and fistulas), which correlated directly with the timing and method of abdominal closure. The authors concluded that the high morbidity rate of 25% is largely dependent on the timing of abdominal closure. The inability to achieve primary abdominal closure is closely associated with large blood transfusion requirements and various extra-abdominal infectious complications; 12% died after wound closure, with seven of these deaths directly related to wound complications [9].

For our patient, it was not possible to close the abdomen after repeated relooks, hence requiring 14 explorations, but fortunately was able to close with the help of mesh repair. These patients are typically critically ill and managed in the intensive care unit early in the disease process.

Early abdomen opening and staged closure are mentioned to be key to better outcomes in abdominal compartment syndrome [6]. The increased length of open abdomen increases the length of ICU and hospital stay, increases multiorgan dysfunction (MODS), fluid loss, lack or improper nutritional supplementation, as well as the surgical complications of enteral fistulas, difficult to close and higher rate of incisional hernias [10].

Manterola et al. evaluated the Bogota bag (BB) as a temporary abdominal closure method [11]. They found that while the technique is an effective and inexpensive way to manage the open abdomen and prevent tension-related complications, it carries a 38% morbidity rate (including enterocutaneous fistulas and surgical-site infections), they also found that the most common reason for the relaparotomy followed by a laparostomy using the Bogota bag was intra-abdominal sepsis (60%), with most patients ultimately developing incisional hernias [11].

The open abdomen has become an important tool for the management of physiologically unstable patients requiring emergent abdominal surgical procedures. These patients present unique challenges to the critical care and nutrition support teams. Careful attention to fluid and electrolyte management, meticulous wound care, prevention of enteroatmospheric fistula, and individualized nutrition support therapy are essential for successful recovery in this patient population [12]. All these patients need intensive care therapy, as these open abdomen patients have repeated bouts of sepsis, hemodynamic instability, and prolonged invasive ventilation. Hence, they will require vasopressor support, fluid and nutritional management, as our patient was complicated by repeated episodes of sepsis and had CINMA, requiring multiple antimicrobials, surgical intervention, and peritoneal lavage, along with optimizing nutritional status with the administration of TPN as well as enteral (naso-jejunal) feeds. The prolonged open abdomen with a number of septic shocks and exploration will increase the duration of invasive ventilation and may require tracheostomy; if the open abdomen is for a shorter duration, the patient may be extubated [13].

## Conclusions

Emergency exploratory laparotomy for intra-abdominal sepsis may result in the inability to close the abdomen, requiring the abdomen to be covered with Bogota bag insertion. Our patient’s abdomen was unable to close due to severe bowel oedema and multiple episodes of intra-abdominal sepsis, requiring 14 laparotomies due to intra-abdominal sepsis and 13 episodes of septic shock. His recovery was further complicated by multiple organ dysfunction syndrome, critical illness neuromuscular abnormalities, and prolonged invasive ventilation requiring tracheostomy. Direct visual impression along with sepsis markers helped in early diagnosis of abdominal sepsis with quicker laparotomy exploration and a quicker recovery from sepsis. In our patient, quicker sepsis assessment and diagnosis, repeated source control, nutritional support (enteral and parenteral) along with chronic intensive care therapy were the key for the favorable outcome.
